# Supplementary material for: The future of antibiotic use in livestock
Source: Nat Commun. 2025 Apr 1;16:2469. doi: 10.1038/s41467-025-56825-7 (PMC11962127; doi:10.1038/s41467-025-56825-7)
Supplement: Supplementary file 1 — Supplementary Information [file 41467_2025_56825_MOESM1_ESM.pdf]

Supplementary Information

Data Sources

Our analysis was based on data from various sources, including Food and Agriculture Organization Corporate Statistics (FAOSTAT)[1], Gridded Livestock of the World version 4.0 (GLW4)[26], Global Livestock Environmental Assessment Model (GLEAM)[2] and the World Organisation for Animal Health (WOAH) Animal Antimicrobial Use Global Database (ANIMUSE)[3]. Our study covers 190 countries across six regions: Asia (58), Africa (54), Europe (40), North America (2), and South America (36). Supplementary Table A1 provides a detailed list of countries for each region. These databases provide information for the analysis, including a detailed and comprehensive understanding of global livestock perspectives, as well as AMU quantities.

This study used the 2019 AMU quantity data from WOAH's seventh annual report on antimicrobial agents intended for use in animals[4]. In addition, we integrated 2019 antimicrobial sales and distribution data from the U.S. FDA[5] and Canada's CIPARS[6] to improve the regional disaggregation of results. The information from WOAH is compiled from reporting countries, involving 157 countries, with 121 providing quantitative information, representing around 85% of WOAH's members and 70% of the global animal biomass. To enhance the reliability of our estimation, we recalibrated the antibiotic intensity by incorporating the latest LBC based biomass estimate considering the percentages attributed to nonreporting countries, as outlined by the WOAH.

The GLW4 dataset is a spatially gridded raster providing detailed information on livestock population densities across spatial units. Specifically, the dataset offers high-resolution spatial information with a grid resolution of 0.083°× 0.083° (radius of ~10 km). GLW4 provides a global, harmonized dataset on livestock distribution, offering population density information for various species such as cattle, buffalo, horses, sheep, goats, pigs, and chickens. The population densities were estimated for each census polygon by dividing the number of animals from the census by the surface area of the administrative unit polygon[7].

Supplementary Table A1: List of countries by region considered in the analysis.

Region	Subregion	Countries
**Asia and the Pacific**	*Central Asia*	Tajikistan, Kyrgyzstan, Uzbekistan, Kazakhstan, Turkmenistan
	Eastern Asia	China, Republic of Korea, Democratic People's Republic of Korea, Mongolia, Japan
	South Eastern Asia	Malaysia, Singapore, Philippines, Timor-Leste, Thailand, Cambodia, Indonesia, Myanmar, Brunei Darussalam, Lao People's Democratic Republic, Viet Nam
	Southern Asia	India, Pakistan, Sri Lanka, Afghanistan, Bangladesh, Nepal, Bhutan, Iran (Islamic Republic of)
	Western Asia	United Arab Emirates, Türkiye, Qatar, Saudi Arabia, Georgia, Oman, Palestine, Cyprus, Armenia, Lebanon, Iraq, Kuwait, Jordan, Israel, Azerbaijan, Syrian Arab Republic, Yemen, Bahrain
	Oceania	Cook Islands, Micronesia, Papua New Guinea, Nauru, New Caledonia, New Zealand, Fiji, Kiribati, Vanuatu, Solomon Islands, Australia
**Africa**	*Eastern Africa*	Rwanda, Ethiopia, Burundi, Djibouti, Uganda, Madagascar, Seychelles, United Republic of Tanzania, Somalia, Mauritius, Mozambique, Zimbabwe, South Sudan, Comoros, Malawi, Kenya, Zambia, Eritrea
	Northern Africa	Morocco, Libya, Sudan, Algeria, Tunisia, Egypt
	Middle Africa	Sao Tome and Principe, Congo, Central African Republic, Democratic Republic of the Congo, Gabon, Equatorial Guinea, Angola, Cameroon, Chad
	Southern Africa	South Africa, Lesotho, Eswatini, Namibia, Botswana
	Western Africa	Mauritania, Niger, Nigeria, Gambia, Guinea, Togo, Sierra Leone, Cabo Verde, Senegal, Mali, Liberia, Guinea-Bissau, Benin, Burkina Faso, Côte d'Ivoire, Ghana
**Europe**	*Northern Europe*	Iceland, Finland, United Kingdom of Great Britain and Northern Ireland, Latvia, Estonia, Sweden, Norway, Faroe Islands, Denmark, Ireland, Lithuania
	Eastern Europe	Belarus, Bulgaria, Ukraine, Republic of Moldova, Poland, Slovakia, Hungary, Romania, Czechia, Russian Federation
	Southern Europe	Spain, Albania, Greece, North Macedonia, Montenegro, Portugal, Croatia, Serbia, Bosnia and Herzegovina, Italy, Malta, Slovenia
	Western Europe	Netherlands, Belgium, Luxembourg, Austria, France, Switzerland, Germany
**Northern Americas**	**Northern America**	United States of America, Canada
**South Americas**	*Mesoamerica*	Bahamas, Guadeloupe, Honduras, Belize, Haiti, Panama, Mexico, Costa Rica, Cuba, Martinique, Grenada, Saint Vincent and the Grenadines, Barbados, Nicaragua, Trinidad and Tobago, Dominica, Antigua and Barbuda, Saint Kitts and Nevis, Jamaica, Puerto Rico, Saint Lucia, Dominican Republic, El Salvador, Guatemala
	South America	Venezuela, Argentina, Ecuador, Uruguay, Chile, Colombia, Brazil, Bolivia, Suriname, Peru, Guyana, Paraguay

Note: The classification of countries into regions and subregions as shown in the table is based on the categorizations used by international organizations such as the Food and Agriculture Organization (FAO), the United Nations (UN), and occasionally by regional development agencies like the World Bank or OECD.

The GLEAM model incorporates disaggregated information on the number of live animals and biomass (Supplementary Figure A1). The model was built based on a life-cycle assessment framework that simulates greenhouse gas emissions in livestock systems, covering various

livestock species, including cattle, sheep, goats, buffaloes, pigs, and chickens across countries. Each species was subdivided into cohorts, and each cohort was associated with the average live weight of the animal[25]. Supplementary Figure A1 presents the herd structure, illustrating geographical regions, animal species, commodity groups, production systems, cohort production cycles, and liveweight information as presented in the GLEAM model.

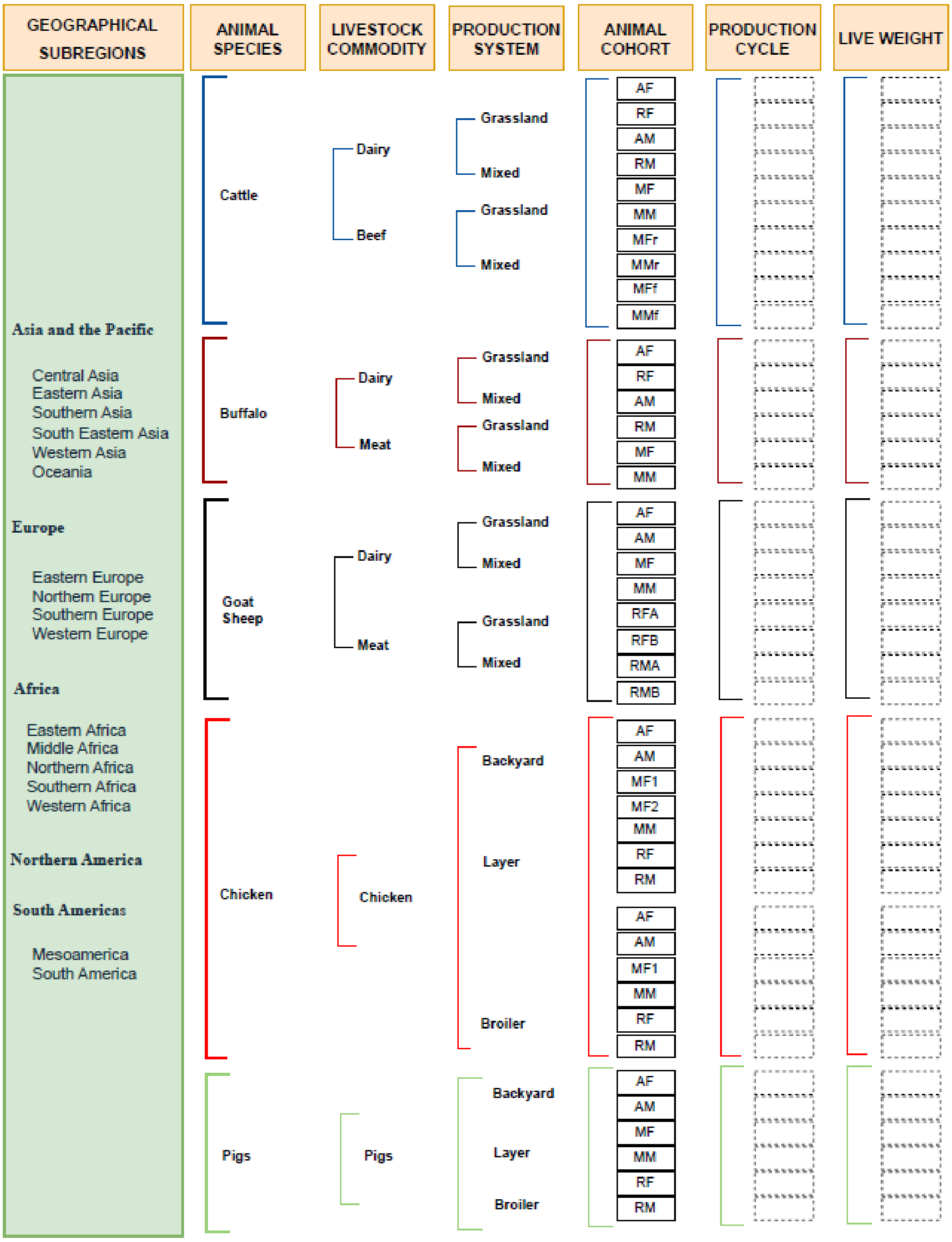

Supplementary Figure.A1 GLEAM Model Herd Structure Disaggregation.

Note: This Supplementary figure illustrates the average live weight of livestock species from the Food and Agriculture Organization (FAO) Global Livestock Environmental Assessment Model (GLEAM) database. The grouping considers the demographic composition of livestock populations across geographical regions, animal species, livestock commodities, production systems, animal cohorts, and production cycles.

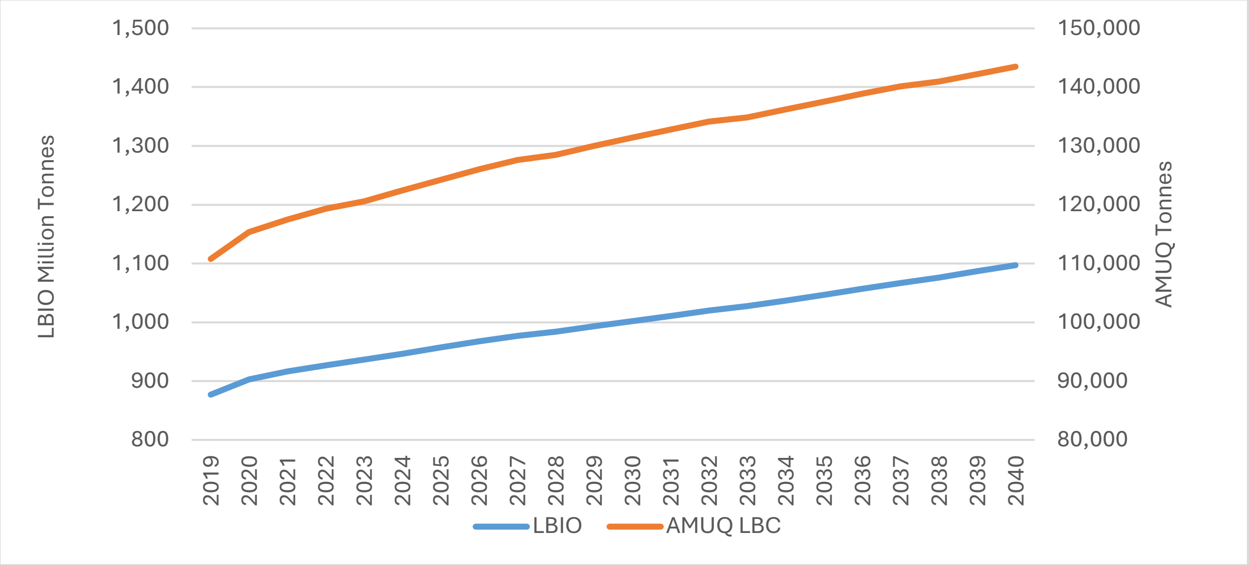

Supplementary Figure.A2 Comparison of LBIO and AMUQ Projections using the LBC Method (2019–2040).

Note: The Supplementary figure presents the global level trends in livestock biomass (LBIO, in million tonnes) and livestock antimicrobial usage quantity (AMUQ) under Livestock Biomass Conversion (LBC, in tonnes) from 2019 to 2040. Both trends indicate an expected increase over the years.

References

1. FAO, F. and A. Organizations. (2023). FAOSTAT - Crops and Livestock Products. https://www.fao.org/faostat/en/
2. FAO. (2022). *Global Livestock Environmental Assessment Model - Version 3* (GLEAM). https://www.fao.org/gleam/resources/en/.
3. WOAH. (2023). Global Database on Animal Antimicrobial Use. Retrieved from https://amu.woah.org/amu-system-portal/home.
4. WOAH. (2023). Seventh Annual Report on Antimicrobial Agents Intended for Use in Animals. Retrieved from https://www.woah.org/app/uploads/2023/05/a-seventh-annual-report-amu-final.pdf
5. U.S. Food and Drug Administration. (2019). *2019 summary report on antimicrobials sold or distributed for use in food-producing animals*. U.S. Department of Health and Human Services. [Available here: https: https://www.fda.gov/media/144427/download]
6. Canadian Integrated Program for Antimicrobial Resistance Surveillance (CIPARS). (2019). CIPARS 2019 annual report. Public Health Agency of Canada. [Available here: https://www.canada.ca/en/public-health/services/surveillance/canadian-integrated-program-antimicrobial-resistance-surveillance-cipars/cipars-2019-integrated-findings.html]
7. Gilbert, M., Nicolas, G., Cinardi, G., Van Boeckel, T. P., Vanwambeke, S. O., Wint, G. R., & Robinson, T. P. (2018). Global distribution data for cattle, buffaloes, horses, sheep, goats, pigs, chickens and ducks in 2010. *Scientific Data,* 5(1), 1-11. https://doi.org/10.1038/sdata.2018.227
